# Supplementary material for: Discrimination of cell-intrinsic and environment-dependent effects of natural genetic variation on Kupffer cell epigenomes and transcriptomes
Source: Nat Immunol. 2023 Sep 21;24(11):1825–38. doi: 10.1038/s41590-023-01631-w (PMC10602851; doi:10.1038/s41590-023-01631-w)
Supplement: Supplementary file 1 — Reporting Summary [file 41590_2023_1631_MOESM1_ESM.pdf]

nature portfolio

Corresponding author(s): Ty D. Troutman and Christopher K. Glass

Last updated by author(s): August 10, 2023

Reporting Summary

Nature Portfolio wishes to improve the reproducibility of the work that we publish. This form provides structure for consistency and transparency in reporting. For further information on Nature Portfolio policies, see our Editorial Policies and the Editorial Policy Checklist.

Statistics

For all statistical analyses, confirm that the following items are present in the figure legend, table legend, main text, or Methods section.

n/a	Confirmed	
☐	☒	The exact sample size (*n*) for each experimental group/condition, given as a discrete number and unit of measurement
☒	☐	A statement on whether measurements were taken from distinct samples or whether the same sample was measured repeatedly
☐	☒	The statistical test(s) used AND whether they are one- or two-sided<br>*Only common tests should be described solely by name; describe more complex techniques in the Methods section.*
☐	☒	A description of all covariates tested
☒	☐	A description of any assumptions or corrections, such as tests of normality and adjustment for multiple comparisons
☐	☒	A full description of the statistical parameters including central tendency (e.g. means) or other basic estimates (e.g. regression coefficient) AND variation (e.g. standard deviation) or associated estimates of uncertainty (e.g. confidence intervals)
☐	☒	For null hypothesis testing, the test statistic (e.g. *F*, *t*, *r*) with confidence intervals, effect sizes, degrees of freedom and *P* value noted<br>*Give P values as exact values whenever suitable.*
☒	☐	For Bayesian analysis, information on the choice of priors and Markov chain Monte Carlo settings
☐	☒	For hierarchical and complex designs, identification of the appropriate level for tests and full reporting of outcomes
☐	☒	Estimates of effect sizes (e.g. Cohen's *d*, Pearson's *r*), indicating how they were calculated

Our web collection on statistics for biologists contains articles on many of the points above.

Software and code

Policy information about availability of computer code

Data collection	Cell sorting data was collected on a MoFlo Astrios EQ and processed with Summit (6.2 and 6.3). Immunoblotting data was collected using ChemiDoc XRS+ System using Image Lab (5.2.1).
Data analysis	Sequencing Data Preprocessing<br>Data was demultiplexed using bcl2fastq (v2.17). RNA-seq was mapped using STAR 2.5. H3K27Ac ChIP-seq and ATAC-seq data were mapped using bowtie4.1.2. ATAC-seq data were trimmed to 30 bp to remove sequencing adapters, for improved mapping efficiency. Strain specific genomes for BALB/cJ and A/J were generated from by replacing invariant positions of mm10 (https://hgdownload.soe.ucsc.edu/goldenPath/mm10/bigZips/) sequence with alleles reported in the Mouse Genome Project strain specific VCF files (ftp://ftp-mouse.sanger.ac.uk/REL-1303-SNPs_Indels-GRCm38/mgp.v3.snps.rsIDdbSNPv137.vcf.gz). mm10 was used as the C57BL/6J strain specific genome. Samples from parental strains of mice were mapped to the strain specific genome. Mapped reads were shifted to the chromosome coordinates of the mm10 genome build using MARGE.pl (v1.0) shift with -ind set to balbcj or aj for reads mapped to the BALB/cJ or A/J genome, respectively.<br><br>For samples from CB6F1/J samples, reads were mapped to the mm10 and BALB/cJ genome builds. Then the BALB/cJ mapped reads were shifted to the mm10 build with MMARGE v1.0 as above. Perfectly mapped reads spanning genetic mutations between BALB/cJ and mm10 were identified using the MMARGE.pl allele_specific_reads command with -ind set to BALB/cJ and a second time with -ind set to mm10 resulting in two SAM files for each biological sample: one SAM file containing reads perfectly mapped to the mm10 genome that spanned known DNA sequence differences relative to the BALB/cJ genome; and a second SAM file containing reads perfectly mapped to the BALB/cJ genome that spanning known DNA sequence differences relative to the reference mm10 genome.<br><br>Tag directories were called with HOMER (4.10) for each tag directory. ATAC-seq peaks were identified with HOMER using relaxed peak finding parameters "-L 0 -C 0 -fdr 0.9 -minDist 200 -size 200". IDR (v2.0.4) was used to test for reproducibility between ATAC-seq replicates. Peaksets

from all pairwise IDR comparisons were merged for further analysis. Feature count matrices RNA-seq were generated using HOMER analyzeRepeats, or using annotatePeaks for ATAC-seq and ChIP-seq.

Statistical Analysis of Sequencing Data
Differential gene expression, histone acetylation, or open chromatin was assessed using DESeq2 v1.16. The raw p-values from DESeq2 for a given peak or gene were corrected for multiple testing using the Benjamini-Hochberg procedure. In some cases, ANOVA was used to assess expression differences of a priori defined candidate genes between many groups. Association of cis/trans with low, equal, and high basal regulation was performed with a Pearson Chi2 test (Python scipy.stats.contingency.chi2_contingency). Python packages used included numpy (1.20.2) and scipy (1.6.2), matplotlib (3.1.1), seaborn (0.9.0). R packages used included bioconductor-edger (3.28.0), gridExtra (2.3), cowplot (1.1.1), tibble (3.2.1), ggfortify (0.4.16), ggplotify (0.1.1), ggrepel (0.9.3) stringr(1.5.0), readr (2.1.4), ggplot2 (3.4.2), patchwork (1.1.2), scales (1.2.1), lubridate (1.9.2), dplyr (1.1.2), tidyr (1.3.0), and tidyverse (2.0.0), ggridges (0.5.4), ggpubr (0.6.0), forcats (1.0.0), stringr (1.5.0), purrr (1.0.1), and tibble (3.2.1)

Maggie Analysis
Maggie (1.2) analysis was performed with modifications to allow analysis of three pairwise comparisons. For each strain, positive sequences (either associated with increased ATAC-seq signal or increased H3K27Ac ChIP-seq signal) were extracted, in addition to the corresponding negative sequence from the comparator strain. Positive and negative sequences from all 6 possible foreground and background strains were concatenated and maximal motifs scores were calculated for each motifs available in JASPAR database and the net difference in max motif score between positive and negative sequences was calculated. Following calculation of net difference in max motif score for all sequences, a non-parametric Wilcoxon signed-rank two-sided test is used to assign significance to putative motifs by comparing the distribution of motif score differences to a null distribution centered on zero.

Niche-net
To assess putative strain-specific ligand activity, we first filtered the NicheNet ligand-target matrix to only consider ligands in which:

1. The ligand was expressed by a cell of the hepatic niche within that strain at > 10 TPM.
2. The receptor was expressed by Kupffer cells from that strain at > 10 TPM.

We also included selected metabolic ligands for which expression data were not available. We did not require that ligands or their receptors be differentially expressed by sender or receiver cells. Target genes were selected to be any gene that had significantly higher expression in a pairwise comparison of that strain (“union” gene set, adjusted p value < 0.05, log fold change > 2, TPM > 10 expression in Kupffer cells). As a background we considered all genes that were expressed at TPM > 10 in Kupffer cells. The NicheNet ligand activity score was then computed as the Pearson correlation coefficient between the ligand-target score and the binary vector indicating whether a target gene was differentially expressed. For heatmaps select top scoring ligands from each strain were aggregated and displayed with ligand z-scores. Ligand receptor interaction scores were displayed for ligand-receptor pairs with a receptor expressed by Kupffer cells in at least one strain. For the circos plot analysis ligand-target interaction scores were displayed as arrow thicknesses linking a ligand to its target gene. R packages used were circlize (0.4.11), dplyr (1.0.2), ggplot2 (3.3.5), tidyr (1.1.2), readr (1.4.0), forcats (0.5.0), stringr (1.4.0), purrr (0.3.4), nichenetr (1.0.0), A.C.Rsuite (1.0.0).

Statistical Analysis of
The effect of strain on NASH CRN and fibrosis score was assessed with a Kruskal-Wallis test (R, Kruskal.test). The effect of NASH or control diets between strains across time on mass was assessed with a linear mixed model fit by maximum likelihood. The hierarchical relationship of repeated measures on individual mice over time was controlled using a random effect term in the model (R, lme4::lmer(Mass ~ 1 + Week * Diet * Genotype + (1 + Week | Mouse), data = df1, REML = F). The significance of main effects or their interactions was assessed using Type III ANOVA with Satterthwaite’s method (R, anova(fittedModel)). Within strain interactions of Week*Diet were assessed by sub-setting data on strain, and running a reduced linear mixed model (R, lmer(Mass ~ 1 + Week * Diet + (1 + Week | Mouse), data = strain_subset, REML = F). R packages used included glmmTMB (1.1.7), nlme (3.1-162), multcomp (1.4-25), tidyverse (2.0.0), sjPlot (2.8.14), lme4 (1.1-34), and lmerTest (3.1.3).

Analysis code is available on GitHub:
https://github.com/HunterBennett/KupfferCell_NaturalGeneticVariation

For manuscripts utilizing custom algorithms or software that are central to the research but not yet described in published literature, software must be made available to editors and reviewers. We strongly encourage code deposition in a community repository (e.g. GitHub). See the Nature Portfolio guidelines for submitting code & software for further information.

Data

Policy information about availability of data

All manuscripts must include a data availability statement. This statement should provide the following information, where applicable:

- Accession codes, unique identifiers, or web links for publicly available datasets
- A description of any restrictions on data availability
- For clinical datasets or third party data, please ensure that the statement adheres to our policy

The datasets generated as part of the current study are available in the Gene Expression Omnibus repository. (GSE216164).

Processed data is made available at Zenodo: https://zenodo.org/record/7829622#.ZHogly-B1qs

Sequencing data was mapped to the publicly available GRCm38/mm10 genome: https://hgdownload.soe.ucsc.edu/goldenPath/mm10/bigZips/

Mouse strains genomes were generated using publicly available Mouse Genomes Project Variant Call Format (VCF) files: ftp://ftp-mouse.sanger.ac.uk/REL-1303-SNPs_Indels-GRCm38/mgp.v3.indels.rsIDdbSNPv137.vcf.gz

Datasets used in figure 3f were sourced from the Gene Expression Omnibus (GEO) Series GSE128337: https://www.ncbi.nlm.nih.gov/geo/query/acc.cgi?acc=GSE128337.

Datasets used in figure 3g were sourced from the Gene Expression Omnibus (GEO) Series GSE128657: https://www.ncbi.nlm.nih.gov/geo/query/acc.cgi?acc=GSE128657.

Human research participants

Policy information about studies involving human research participants and Sex and Gender in Research.

Reporting on sex and gender	Not applicable
Population characteristics	Not applicable
Recruitment	Not applicable
Ethics oversight	Not applicable

Note that full information on the approval of the study protocol must also be provided in the manuscript.

Field-specific reporting

Please select the one below that is the best fit for your research. If you are not sure, read the appropriate sections before making your selection.

☒ Life sciences ☐ Behavioural & social sciences ☐ Ecological, evolutionary & environmental sciences

For a reference copy of the document with all sections, see nature.com/documents/nr-reporting-summary-flat.pdf

Life sciences study design

All studies must disclose on these points even when the disclosure is negative.

Sample size	Sample size calculations were performed using a power analysis method detailed in Hart et al., J Comp Bio, 2014. According to these calculations a minimum of 2 samples would allow detection of 2-fold changes with >90% power. Therefore RNA-seq and ATAC-seq, and H3K27Ac ChIP-seq studies used a minimum of two biological replicates per cell subset.<br><br>We determined effect sizes of our own pathological scoring data from mouse NASH experiments and determined that we had >90% power to detect a 2-fold change in NASH CRN score at $\alpha = 0.05$, and 43% power to detect a 60% fold change in fibrosis score at $\alpha = 0.05$.
Data exclusions	Data quality was assessed using Spearman correlation between replicates Spearman correlation was calculated using TPM values for RNA-seq data, and tags overlying ATAC-seq peaks for ATAC-seq and H3K27Ac ChIP-seq data. Correlation was subset per strain, cell type, and condition and only replicates with good correlation (>0.80 typically, but determined on a case-by-case basis) were kept.
Replication	RNA-seq, ATAC-seq, and H3K27Ac ChIP-seq in Kupffer cells other non-parenchymal cell types from the inbred strains and CB6F1/J hybrids were performed in 2 independent experiments with highly correlated findings. All assays were successfully replicated 2 or more times and quantification and statistics are run on combined replicate experiments.<br><br>AMLN diet data included data from >5 replicated experiments.<br><br>RNA-seq from hepatocytes isolated from A/J, BALB/cJ, and C57BL/6J mice had n=2 independent samples but was not independently replicated as this data was only used for identifying putative ligands for use in the NicheNet algorithm.
Randomization	Data was generated from littermate mice ordered directly from the Jackson laboratories. Mice from the same shipment and, when possible, the same cage, were used within experimental groups. Littermates were assigned randomly for treatment with lipopolysaccharide or phosphate buffered saline. When assessing response of each strain to a NASH inducing diet, littermates were split randomly into separate cages and subsequently fed a NASH inducing diet or custom control diet for 30 weeks.
Blinding	Blinding was used in the assessment of liver pathology, which was performed by a board-certified pathologist. In this case the pathologist was given histopathologic slides with encoded IDs that were scored using the NASH CRN and fibrosis scores. These encoded IDs were then translated back to the original sample ID by the research team. Researchers were not blinded to groupings for other experiments as mice received identical treatments.

Reporting for specific materials, systems and methods

We require information from authors about some types of materials, experimental systems and methods used in many studies. Here, indicate whether each material, system or method listed is relevant to your study. If you are not sure if a list item applies to your research, read the appropriate section before selecting a response.

Materials & experimental systems

n/a	Involved in the study
☐	☒ Antibodies
☒	☐ Eukaryotic cell lines
☒	☐ Palaeontology and archaeology
☐	☒ Animals and other organisms
☒	☐ Clinical data
☒	☐ Dual use research of concern

Methods

n/a	Involved in the study
☐	☒ ChIP-seq
☐	☒ Flow cytometry
☒	☐ MRI-based neuroimaging

Antibodies

Antibodies used

Anti-Mouse/Human CD11b PE/Cy7 (clone M1/70); BioLegend; 101216; RRID: AB_312799
Anti-Mouse CD146 BUV395 (clone ME-9F1); BD Biosciences; 740330; RRID: AB_2740063
Anti-Mouse CD16/32 (clone 93); BioLegend; 101302; RRID: AB_312801
Anti-Mouse CD45 Alexa488 (clone 30F11); BioLegend; 103122; RRID: AB_493531
Anti-Mouse CD45 BB515 (clone 30-F11); BD Biosciences; 564590; RRID: AB_2738857
Anti-Mouse Cx3cr1 Alex647 (clone SA011F11); BioLegend; 149004; RRID: AB_2564273
Anti-Mouse F4/80 BV421 (clone BM8); BioLegend; 123132; RRID: AB_11203717
Anti-Mouse Tim4 Alexa647 (clone RMT4-54); BioLegend; 127641; RRID: AB_2271648
Anti-Mouse Tim4 PE (clone RMT4-54); BioLegend; 130008; RRID: AB_2201843
Anti Mouse H-2Dd PE (clone 34-2-12); BioLegend; 110607; RRID: AB_313488
Anti Mouse H-2Kb Alexa647 (clone AF6-88.5); BioLegend; 116512 ; RRID: AB_492917
Anti-phospho-STAT3 (Tyr705) (clone D3A7); Cell Signaling Technology; 9145; RRID: AB_2491009
Anti-STAT3 (clone 124H6); Cell Signaling Technology; 9139; RRID:AB_331757
Donkey Anti-Mouse Immunoglobulins/HRP (Dako); no longer available on the manufacturer's website
Donkey Anti-Rabbit Immunoglobulins/HRP (Dako); no longer available on the manufacturer's website

Validation

Anti-Mouse/Human CD11b PE/Cy7 - validated by manufacturer in flow assays and internally using fluorescence minus one controls
Anti-Mouse CD146 BUV395 - validated by manufacturer in flow assays and internally using fluorescence minus one controls
Anti-Mouse CD16/32 - validated by manufacturer in flow assays
Anti-Mouse CD45 Alexa488 - validated by manufacturer in flow assays and internally using fluorescence minus one controls
Anti-Mouse CD45 BB515 - validated by manufacturer in flow assays and internally using fluorescence minus one controls
Anti-Mouse Cx3cr1 Alexa647 - validated by manufacturer in flow assays and internally using fluorescence minus one controls
Anti-Mouse F4/80 BV421 - validated by manufacturer in flow assays and internally using fluorescence minus one controls
Anti-Mouse Tim4 Alexa647 - validated by manufacturer in flow assays and internally using fluorescence minus one controls
Anti-Mouse Tim4 PE - validated by manufacturer in flow assays and internally using fluorescence minus one controls
Anti Mouse H-2Dd PE - validated by manufacturer in flow assays and internally using fluorescence minus one controls
Anti Mouse H-2Kb Alexa647 - validated by manufacturer in flow assays and internally using fluorescence minus one controls
Anti-phospho-STAT3 - validated by manufacturer in immunoblotting assays
Anti-STAT3 - validated by manufacturer in immunoblotting assays

Detailed validation information is available on the product specification sheets.

Animals and other research organisms

Policy information about studies involving animals; ARRIVE guidelines recommended for reporting animal research, and Sex and Gender in Research

Laboratory animals

Mus musculus laboratory mice were used in all experiments. Baseline studies were performed with male A/J, BALB/cJ, C57BL/6J and CB6F1/J (first generation intercross of male C57BL/6J and female BALB/cJ) mice age 8-10 weeks.

NOD-scid IL2Rgnull mice age 8-10 weeks were conditioned with the myeloablative agent busulfan at 25 mg/kg for 2 consecutive days. On the third day, mice were engrafted via retro orbital injection with magnetically enriched, lineage negative, hematopoietic stem cells (Miltenyi) from BALB/cJ, C57BL/6J, or CB6F1/J donors. Engraftment efficiency was monitored in peripheral blood after 4 and 8 weeks.

Mice were fed for up to 30 weeks with a NASH-model diet (Research Diets, D09100301) composed of 40 kcal% from fat, 20 kcal% from fructose, and 2% cholesterol by mass, or a custom defined control diet (Research Diet, D15100601) composed of 10% kcal from fat with 50 g inulin (a dietary fiber) per 4,057 kcal.

Mice were housed in individually ventilated cages in standard conditions at 22°C with 40 ± 5% relative humidity and a 12-h light/dark cycle. Water and standard laboratory diet were available ad libitum, unless indicated otherwise.

Wild animals

Study did not involve wild animals.

Reporting on sex

Sex was not considered as a variable in these studies due to poor susceptibility of female mice of the chosen strains to NASH models.

Field-collected samples

The study did not involve samples collected from the field.

Ethics oversight | All animals were maintained and all procedures performed were approved by the University of California San Diego Animal Care and Use Committee in accordance with an approved animal study protocol meeting AALAC standards.

Note that full information on the approval of the study protocol must also be provided in the manuscript.

ChIP-seq

Data deposition

☒ Confirm that both raw and final processed data have been deposited in a public database such as GEO.

☒ Confirm that you have deposited or provided access to graph files (e.g. BED files) for the called peaks.

Data access links
May remain private before publication.

Sequencing data generated in this study are available at GEO through accession number GSE216164.

Graph files (HOMER peaks) are available on GEO as well as Zenodo:
https://zenodo.org/record/7829622#.ZHogly-B1qs

Files in database submission

h3k27ac_AJ_Kupffer_Control_rep1_R1.fastq.gz
h3k27ac_AJ_Kupffer_Control_rep1_R2.fastq.gz
h3k27ac_AJ_Kupffer_Control_rep2_R1.fastq.gz
h3k27ac_AJ_Kupffer_Control_rep2_R2.fastq.gz
h3k27ac_AJ_Kupffer_Control_rep3_R1.fastq.gz
h3k27ac_AJ_Kupffer_Control_rep3_R2.fastq.gz
h3k27ac_BALBcJ_Kupffer_Control_rep1_R1.fastq.gz
h3k27ac_BALBcJ_Kupffer_Control_rep1_R2.fastq.gz
h3k27ac_BALBcJ_Kupffer_Control_rep2_R1.fastq.gz
h3k27ac_BALBcJ_Kupffer_Control_rep2_R2.fastq.gz
h3k27ac_BALBcJ_Kupffer_Control_rep3_R1.fastq.gz
h3k27ac_BALBcJ_Kupffer_Control_rep3_R2.fastq.gz
h3k27ac_C57_Kupffer_Control_rep1_R1.fastq.gz
h3k27ac_C57_Kupffer_Control_rep1_R2.fastq.gz
h3k27ac_C57_Kupffer_Control_rep2_R1.fastq.gz
h3k27ac_C57_Kupffer_Control_rep2_R2.fastq.gz
h3k27ac_C57_Kupffer_Control_rep3_R1.fastq.gz
h3k27ac_C57_Kupffer_Control_rep3_R2.fastq.gz
h3k27ac_cb6f1j_kupffer_chow_rep1_R1.fastq.gz
h3k27ac_cb6f1j_kupffer_chow_rep1_R2.fastq.gz
h3k27ac_cb6f1j_kupffer_chow_rep2_R1.fastq.gz
h3k27ac_cb6f1j_kupffer_chow_rep2_R2.fastq.gz
h3k27ac_cb6f1j_kupffer_chow_rep3_R1.fastq.gz
h3k27ac_cb6f1j_kupffer_chow_rep3_R2.fastq.gz
input_AJ_Kupffer_Control_rep1_R1.fastq.gz
input_AJ_Kupffer_Control_rep1_R2.fastq.gz
input_AJ_Kupffer_Control_rep2_R1.fastq.gz
input_AJ_Kupffer_Control_rep2_R2.fastq.gz
input_AJ_Kupffer_Control_rep3_R1.fastq.gz
input_AJ_Kupffer_Control_rep3_R2.fastq.gz
input_BALBcJ_Kupffer_Control_rep1_R1.fastq.gz
input_BALBcJ_Kupffer_Control_rep1_R2.fastq.gz
input_BALBcJ_Kupffer_Control_rep2_R1.fastq.gz
input_BALBcJ_Kupffer_Control_rep2_R2.fastq.gz
input_BALBcJ_Kupffer_Control_rep3_R1.fastq.gz
input_BALBcJ_Kupffer_Control_rep3_R2.fastq.gz
input_C57_Kupffer_Control_rep1_R1.fastq.gz
input_C57_Kupffer_Control_rep1_R2.fastq.gz
input_cb6f1j_kupffer_chow_rep1_R1.fastq.gz
input_cb6f1j_kupffer_chow_rep1_R2.fastq.gz
input_cb6f1j_kupffer_chow_rep2_R1.fastq.gz
input_cb6f1j_kupffer_chow_rep2_R2.fastq.gz
input_cb6f1j_kupffer_chow_rep3_R1.fastq.gz
input_cb6f1j_kupffer_chow_rep3_R2.fastq.gz

h3k27ac_f0_raw.txt
h3k27ac_f1_raw.txt

Genome browser session
(e.g. UCSC)

https://genome.ucsc.edu/s/hunterrb/glass_kupffer_strains_manuscript

Methodology

Replicates | N=3 independent replicates were used for each experimental subgroup. Samples with pearson correlation of H3K27Ac ChIP-seq signal annotated over ATAC-seq peaks >0.8 were kept for analysis.

Sequencing depth	Inbred strain samples were sequenced to a depth of ~5 million reads. F1 samples were sequenced to a depth of ~50 million reads to allow sufficient detection of perfectly mapped reads spanning mutations between the parental genomes.
Antibodies	Active Motif, 39685, anti-H3K27ac, RRID: AB_2793305, Clone: MABI 0309
Peak calling parameters	Our pipeline did not call H3K27Ac ChIP-seq peaks, instead ChIP-seq signal was annotated over relevant ATAC-seq peaks identified using the irreproducible discovery rate (IDR v2.0) algorithm. IDR ATAC-seq peaks were annotated using HOMER (v4.10) annotate_peaks command using -size 1000 flag to set a 2000bp window around the ATAC-seq peak.
Data quality	Data were examined on the UCSC genome browser to ensure efficacy of the immunoprecipitation step. Outlier samples were screened for using Spearman correlation between samples within each subgroup. All samples with a Spearman correlation > 0.90 were retained for further analysis.
Software	H3K27Ac ChIP-seq was mapped to strain specific genomes (mm10 was used as the C57BL/6J strain specific genome) generated from Mouse Genome Project VCF files. Mapping was performed using bowtie 0.12.7. Samples from parental strains of mice were mapped to the strain specific genome. Mapped reads were shifted to the chromosome coordinates of the mm10 genome build using MARGE.pl (v1.0) shift with -ind set to balbcj or aj for reads mapped to the BALB/cJ or A/J genome, respectively. Tag directories were generated from SAM alignment files using HOMER v4.10.

Flow Cytometry

Plots

Confirm that:

☒ The axis labels state the marker and fluorochrome used (e.g. CD4-FITC).

☒ The axis scales are clearly visible. Include numbers along axes only for bottom left plot of group (a 'group' is an analysis of identical markers).

☒ All plots are contour plots with outliers or pseudocolor plots.

☒ A numerical value for number of cells or percentage (with statistics) is provided.

Methodology

Sample preparation	Mouse livers were retrograde perfused for 3 min at a rate of 5-7 mL/min through the inferior vena cava with HBSS without Ca2+ or Mg2+ supplemented with 0.5 mM EGTA, 0.5 mM EDTA, and 20 mM HEPES. Perfusions were then switched to 40 mL of a digestion buffer, held at 37°C , comprised of HBSS with Ca2+ and Mg2+ supplemented with 0.033 mg/mL of Liberase TM (Roche), 20 mg/mL DNaseI (Worthington), and 20 mM HEPES. Livers were then excised, minced, and digested for an additional 20 min in vitro at 37°C with gentle rotation in 20 mL of fresh digestion buffer. The perfusion and digestion steps were performed in the presence of 1 mM flavopiridol to offset transcriptional changes associated with digestion. After tissue digestion, cells were passed through a 70-micron cell strainer and hepatocytes removed by 2 low-speed centrifugation steps at 50 X G for 2 min. Non-parenchymal cells in the supernatant were further separated from debris by pelleting for 15 min at 600 X G in 50 mL of 20% isotonic Percoll (Sigma Aldrich) at room temperature. Cells were then washed from Percoll containing buffer and suspended in 10 mL 28% OptiPrep (Sigma Aldrich) and carefully underlaid beneath 3 mL of wash buffer. The resulting gradient was centrifuged at 1,400 X G for 25 min at 4°C with no break, and cells enriched at the interface were saved and subjected to isotonic erythrocyte lysis. Cells were washed after erythrocyte lysis and immediately used purified by cells sorting.
Instrument	Beckman Coulter Mo-Flo Astrios EQ configured with spatially separated 355 nm, 405 nm, 488 nm, 561 nm, and 642 nm lasers
Software	The Astrios was controlled and set up using Summit software. Some post-sort analyses were done using FlowJo (10.4.1).
Cell population abundance	Post-sort purities routinely exceeded 95% as assessed by reacquisition on the same cell sorter. Transcriptomics is sensitive to contamination and we could observe indications that our method for purifying hepatic stellate cells had varied contamination with Kupffer cells.
Gating strategy	Kupffer cells were defined as 355:494/20Low (inferred as retinol autofluorescence), SSCLow, CD146Neg, CD45Pos, F4/80High, CD11bIntermediate, Live, Singlets. Liver sinusoidal endothelial cells were defined at 355:494/20Low (inferred as retinol autofluorescence), SSCLow, CD45Neg, CD146Pos, Live, Singlets. Hepatic stellate cells were defined as 355:494/20High (inferred as retinol autofluorescence), SSCIntermediate, Live, Singlets. Singlets were identified using SSC-H/SSC-A and FSC-H/FSC-A for all populations.

☒ Tick this box to confirm that a figure exemplifying the gating strategy is provided in the Supplementary Information.
