# Supplementary figures and images for: Discrimination of cell-intrinsic and environment-dependent effects of natural genetic variation on Kupffer cell epigenomes and transcriptomes
Source: Nat Immunol. 2023 Sep 21;24(11):1825–38. doi: 10.1038/s41590-023-01631-w (PMC10602851; doi:10.1038/s41590-023-01631-w)

Figure 3e

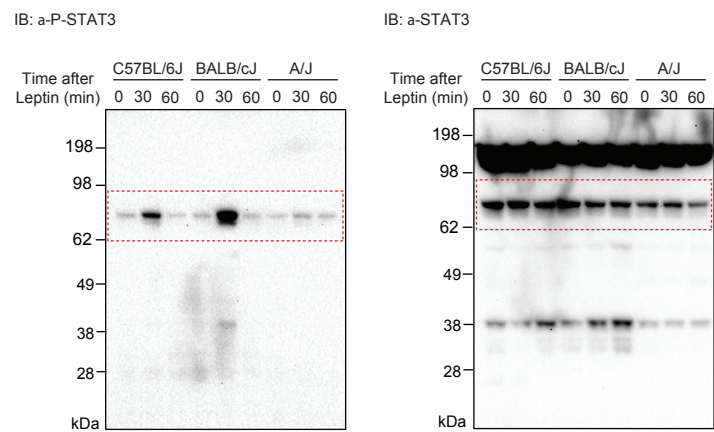

Uncropped images of immunoblots

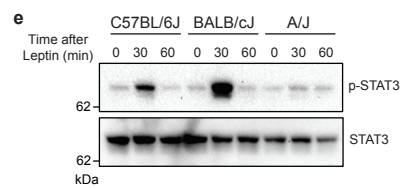

Cropped version in Figure 3e

Supplement: Supplementary file 9 — Unprocessed immunoblot images for Fig. 3e. [file 41590_2023_1631_MOESM9_ESM.pdf]
